# Linking phytochemistry to bioactivity: *in vitro, in vivo*, and *in silico* insights into Rubia tinctorum

**DOI:** 10.3389/fchem.2026.1847787

**Published:** 2026-06-24

**Authors:** Fatima Zahra Ayyad, Mouad Lahyaoui, Mohammed Al-zharani, Chaimae Ibbur, Mohamed Radi, Aicha Benouahi, Morad Kaddouri, Aziz Drioiche, Fahd A. Nasr, Ashraf Ahmed Qurtam, Ahde El Imache, Touriya Zair

**Affiliations:** 1 Research Team of Chemistry of Bioactive Molecules and the Environment, Laboratory of Innovative Materials and Biotechnology of Natural Resources, Faculty of Sciences, Moulay Ismaïl University, Meknes, Morocco; 2 Laboratory of Innovative Technologies, Process Engineering Department, Higher School of Technology Fez, Sidi Mohamed Ben Abdellah University, Fez, Morocco; 3 Laboratory of Applied Organic Chemistry, Faculty of Science and Technology, Sidi Mohamed Ben Abdellah University, USMBA, Fez, Morocco; 4 Biology Department, College of Science, Imam Mohammad Ibn Saud Islamic University (IMSIU), Riyadh, Saudi Arabia; 5 Laboratory of the Engineering and Applied Technologies, Higher School of Technology, Sultan Moulay Slimane University, Beni Mellal, Morocco; 6 Higher Institute of Nursing and Health Techniques of Fez, Regional Health Directorate Fez-Meknes, EL Ghassani Hospital, Fez, Morocco

**Keywords:** antihyperglycemic, antimicrobial, antioxidant, *in silico*, *Rubia tinctorum*, toxicity

## Abstract

**Introduction:**

This study investigated the phytochemical composition and biological activities of the aqueous root extract of *Rubia tinctorum* L. using *in vitro, in vivo*, and *in silico* approaches.

**Methods:**

Phenolic compounds were extracted by decoction and quantified as total polyphenols, flavonoids, and condensed tannins. The chemical profile of the extract was characterized using HPLC/UV-ESI-MS. Antioxidant activity was evaluated using total antioxidant capacity (TAC), DPPH radical scavenging, and ferric reducing antioxidant power (FRAP) assays, while antimicrobial activity was assessed against selected bacterial and fungal strains. Acute toxicity and antihyperglycemic effects were investigated *in vivo*, and molecular docking explored interactions between the extract’s major compounds and selected biological targets.

**Results:**

The extract contained 40.58 ± 1.01 mg GAE/g DE, 9.43 ± 0.81 mg QE/g DE, and 11.96 ± 0.45 mg CE/g DE of total polyphenols, flavonoids, and condensed tannins, respectively. HPLC/UV-ESI-MS revealed the presence of ruberythric acid, galiosin, rubiadin, salvianolic acid C, and pseudopurpurin glucoside as major compounds. The extract exhibited antioxidant activity (IC_50_ = 265.17 ± 0.68 μg/mL for DPPH; EC_50_ = 147.04 ± 0.71 μg/mL for FRAP) and a TAC of 165.26 ± 0.46 mg AAE/g DE. Significant antimicrobial activity was observed against *Escherichia coli, Proteus mirabilis, Saccharomyces cerevisiae, Aspergillus niger*, and several *Candida* strains. In addition, the extract exhibited notable antihyperglycemic activity without inducing acute toxicity. Molecular docking revealed strong binding affinities of ruberythric acid and salvianolic acid C toward key protein targets.

**Discussion:**

These findings highlight the significant antioxidant, antimicrobial, and antihyperglycemic potential of the extract.

## 1 Introduction

Phytotherapy continues to play an essential role in global healthcare systems (Idm’hand et al., 2020). According to the World Health Organization (WHO), approximately 80% of the population in developing countries relies on medicinal plants for the treatment of various diseases (Ait Bouzid et al., 2023). Their extensive use in pharmaceutical, medical, nutritional, and cosmetic applications is largely attributed to their accessibility, affordability, and richness in bioactive secondary metabolites such as polyphenols, flavonoids, and alkaloids (Ait Bouzid et al., 2023; Alli et al., 2011).

Morocco possesses remarkable floristic diversity due to its varied geographical and climatic conditions, hosting approximately 4,500 plant species distributed across 940 genera and 135 families (Amrati et al., 2021; Chaachouay et al., 2022; El Alami et al., 2016). Many Moroccan medicinal plants are widely used in traditional pharmacopoeia and represent valuable sources of bioactive compounds capable of mitigating oxidative stress–related disorders, including cardiovascular, neurodegenerative, and metabolic diseases (Bouyahya et al., 2021; Gohari et al., 2011; Narayanaswamy and Balakrishnan, 2011).

Among these species, *Rubia tinctorum* L (Rubiaceae), commonly known as madder, is a perennial medicinal plant traditionally used in several regions of the world (Cardon, 2014). In Morocco, it grows naturally in the Rif, Middle Atlas, High Atlas, Beni Snassen, and Debdou Mountains, reaching altitudes of up to 2,000 m (Aafi et al., 2002). Traditionally, crude extracts of *R. tinctorum* roots have been used for their anti-inflammatory, antibacterial, and antifungal properties, as well as for the management of anemia, jaundice, urinary stones, constipation, liver disorders, and diarrhea (Bellakhdar, 1997; Lajkó et al., 2015; Marhoume et al., 2019; ODOUNGA, 2011).

Previous *in vitro* and *in vivo* investigations have demonstrated a wide range of biological activities for *R. tinctorum*, including antioxidant, anticancer, anti-inflammatory, antimicrobial, hepatoprotective, neuroprotective, diuretic, and antiparasitic effects. These pharmacological properties are mainly attributed to the presence of bioactive anthraquinones and related phenolic compounds in the roots and rhizomes, such as alizarin, purpurin, pseudopurpurin, purpuroxanthin, ruberythric acid, munjistin, rubiadin, nordamnacanthal, and lucidin primeveroside (El-Banna et al., 2025; Lajkó et al., 2015; Marhoume et al., 2019; Ohta et al., 2025).

Despite these promising findings, comprehensive studies integrating phytochemical profiling, biological evaluation, and computational approaches remain limited, particularly for Moroccan populations of *R. tinctorum* roots. Therefore, the present study aimed to investigate the phytochemical composition and biological activities of *R. tinctorum* root aqueous extract. The study included the determination of phenolic contents, chemical characterization using HPLC/UV-ESI-MS, and evaluation of antioxidant, antimicrobial, and antihyperglycemic activities, together with acute toxicity assessment. In addition, molecular docking analysis was performed to predict the interactions between major phytochemicals and key protein targets associated with oxidative stress, microbial resistance, and glucose metabolism, thereby providing deeper insight into the potential mechanisms underlying the biological activities of this medicinal plant.

## 2 Materials and methods

### 2.1 Materials

#### 2.1.1 Plant material

**FIGURE 1:**
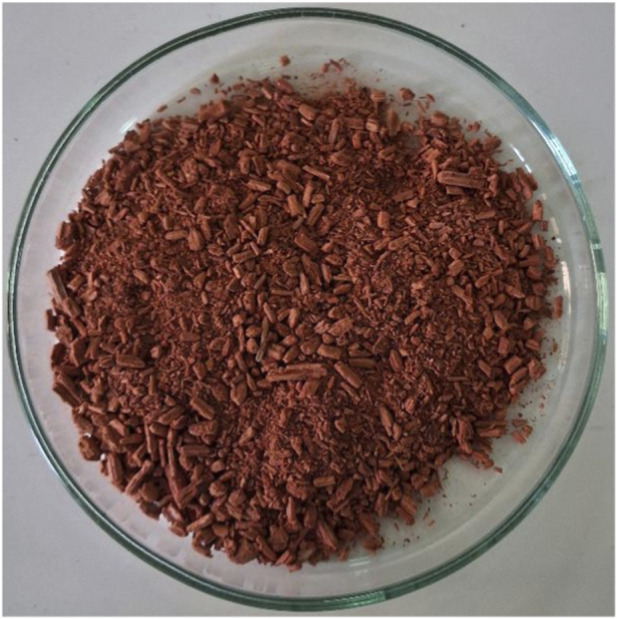
Chopped dried roots of *Rubia tinctorum* L.

*Rubia tinctorum* L (Rubiaceae*)*, commonly known as dyer’s madder, was collected in April 2024 from Ksar Flilou, Midelt region, Morocco (32.6295° N, 4.7529° W). The plant material was taxonomically identified by Professor Amina Bari, botanist at the Biology Department, Faculty of Sciences Dhar El Mahraz (FSDM), Sidi Mohamed Ben Abdellah University (USMBA), Fez, Morocco. A voucher specimen was deposited in the herbarium of the Biology Department under the reference number RRT0010424M. The roots were cleaned, shade-dried at ambient temperature until complete dehydration, and then ground into a fine powder using a laboratory grinder (Figure 1). The powdered material was stored in airtight containers in a cool and dark place at room temperature until further analysis.

#### 2.1.2 Microbial material

Nine bacterial strains and eight fungal strains were used to evaluate the antimicrobial activity of *R. tinctorum* root aqueous extract (Table 1). The bacterial strains were isolated from the Mohamed V Provincial Hospital in Meknes, Morocco, while the fungal strains, including yeasts, were obtained from the Mycotheca of the Parasitology-Mycology Laboratory at the Ibn Sina Hospital Center (ISHC), Rabat, Morocco. All isolates were previously identified using the automated BD Phoenix system for identification and antibiotic susceptibility testing. The microorganisms were stored at −80 °C in 20% glycerol until use. Prior to experimentation, strains were reactivated on Mueller–Hinton agar for bacteria and Sabouraud agar for fungi, followed by subculturing to ensure viability and purity. The selected microorganisms are well known for their pathogenicity, invasiveness, and high antimicrobial resistance, representing major therapeutic challenges in Morocco. *Staphylococcus aureus primarily* causes skin infections; *Escherichia coli* is associated with urinary tract infections; *Klebsiella pneumoniae* is linked to septicaemia, pneumonia, endocarditis, and endogenous endophthalmitis; and *Pseudomonas aeruginosa* is associated with respiratory infections (Nortjie et al., 2022).

**TABLE 1:** List of bacterial and fungal strains tested.

Bacterial strains	Abbreviations	Fungal strains	Abbreviations
*Staphylococcus epidermidis*	*S. epidermidis*	*Candida albicans*	*C. albicans*
*Staphylococcus aureus* BLACT	*S. aureus* BLACT	*Candida kefyr*	*C. kefyr*
*Streptococcus agalactiae* (B)	*S. agalactiae* (B)	*Candida krusei*	*C. krusei*
*Escherichia coli* ESBL	*E. coli* ESBL	*Candida parapsilosis*	*C. parapsilosis*
*Klebsiella pneumonie*	*K. pneumonie*	*Candida tropicalis*	*C. tropicalis*
*Escherichia coli*	*E. coli*	*Candida dubliniensis*	*C. dubliniensis*
*Proteus mirabilis*	*P. mirabilis*	*Saccharomyces cerevisiae*	*S. cerevisiae*
*Pseudomonas aeruginosa*	*P. aeruginosa*	*Aspergillus niger*	*A. niger*
*Enterobacter cloacae*	*E. cloacae*	​	​

#### 2.1.3 Animals

Acute toxicity study was conducted on albino mice (both male and female, body weight 20–35 g), while the *in vivo* antihyperglycemic activity assessment was performed using Wistar rats (both male and female, body weight 200–250 g). All animals were rared under standard laboratory conditions, with a 12 h light/12 h dark phtoperiod, a controlled temperature of 22 °C ± 2 °C, and free access to food and water. They were housed in polycarbonate cages (21.5 × 46.5 × 14.5 cm) with wire tops and wood shavings bedding, which was replaced once or twice weekly. All experimental procedures and protocols were performed in accord with ethical guidelines and regulations to ensure animal welfare, with the study being reviewed and approved by the Institutional Animal Care and Use Committee (IACUC) of laboratory Animals at the Faculty of Sciences Dhar El Mehraz, Sidi Mohamed Ben Abdallah University, Fez, Morocco (10/04/2019/LBEAS) (Albus, 2012).

### 2.2 Methods

#### 2.2.1 Quality control of plant material of *Rubia tinctorum* roots

##### 2.2.1.1 pH determination

This method consists mixing 2 g of plant material with 10 mL of hot distilled water. After 5 min of stirring at room temperature, the mixture was filtered, cooled. The pH was measured by immersing the pH meter electrode in a sufficient volume of the filtrate (Hayani et al., 2022).

##### 2.2.1.2 Moisture content

Moisture content (MC) was determined according to the AFNOR standard (NF-V03-402, 1985), comparing the mass of the plant before and after drying. A 5 g portion of the air-dried plant was put in Petri dishes, and oven-dried at 100 °C ± 5 °C for 24 h (NFV03-402, 1985). The Petri dishes were then cooled in a desiccator before being weighed again. The moisture content was determined using the following Equation 1.

$$MC\%=\left(\frac{m_{0}-m_{1}}{m_{0}}\right)\times 100$$ (1)

where m_0_: Initial mass of the plant (g); m_1_: Mass after drying (g).

##### 2.2.1.3 Ash content

The ash content represents the mineral residue that remains after the combustion of organic matter (OM) at high temperatures in a muffle furnace. Following the AFNOR standard (NF ISO 5984) (NFV05-113, 1972), a 2 g portion of the ground plant material was incinerated at 550 °C until all organic particles were eliminated, yielding a constant mass of white ash. The organic matter content was calculated using the following Equation 2:

$$OM\%=\left(\frac{m_{1}-m_{2}}{TS}\right)\times 100$$ (2)

m_1_: Mass of the capsule and sample before calcination (g);

m_2_: Mass of the capsule and sample after calcination (g);

TS: Test sample (g).

The ash content was calculated using Equation 3:

Ash%=100−OM% (3)

##### 2.2.1.4 Dosage of trace metals by ICP-AES

Inductively coupled plasma atomic emission spectrometry (ICP-AES; Ultima 2, Jobin Yvon, HORIBA, Stow, MA, United States) was employed to determine the concentrations of trace metals. The standard mineralization protocol (AFNOR, 1999) using aqua regia was followed to quantify key elements (As, Zn, Co., Mn, Fe, Cu, and Al). Briefly, 0.1 g of finely crushed plant material was mixed with 3 mL of aqua regia, consisting of 1 mL of concentrated nitric acid (HNO_3_; 99%) and 2 mL of hydrochloric acid (HCl; 37%). The mixture was heated under reflux at 200 °C for 2 h, cooled, and allowed to settle. The supernatant was collected, filtered through a 0.45 µm membrane, and diluted to a final volume of 15 mL with distilled water (Skujins, 1998). ICP-AES analysis was performed at the UATRS laboratory (Technical Support Unit for Scientific Research) of the CNRST in Rabat.

#### 2.2.2 Phytochemical screening

Phytochemical screening was carried out to identify primary and secondary metabolites in *R. tinctorum* roots, providing preliminary insight into the chemical profile. This screening was carried out according to standard colorimetric and precipitation tests: lipids (plant powder, identified by Liebermann–Burchard test); polysaccharides, proteins, and reducing sugars (5% aqueous decoction, iodine, Biuret/xanthoproteic, and Fehling’s tests, respectively); sterols and triterpenes (10% ether macerate, Liebermann–Burchard test); flavonoids (5% aqueous infusion, cyanidin reaction); tannins (5% aqueous infusion, total tannins by FeCl_3_, catechic and gallic tannins by Stiasny reagent); anthocyanins and leucoanthocyanins (5% aqueous infusion, HCl and isoamyl alcohol tests); saponins (1% aqueous decoction, foam test); monosaccharides and holosides (10% aqueous decoction, Barfoed’s test); alkaloids (10% H_2_SO_4_ macerate, Dragendorff and Wagner reagents); reducing compounds (10% aqueous decoction, Fehling’s test); and mucilages (10% aqueous decoction, alcohol precipitation) (Joshi et al., 2013; Pandey and Tripathi, 2014; Shaikh and Patil, 2020).

#### 2.2.3 Extraction of phenolic compounds

The aqueous extract of *R. tinctorum* roots was prepared by decoction according to a previously described method (Saidi et al., 2023). Briefly, 30 g of powdered root material was mixed with 600 mL of distilled water and heated at 80 °C under continuous stirring for 60 min. The resulting mixture was filtered through Whatman filter paper to remove plant residues. The filtrate was subsequently dried in an oven at 70 °C until complete solvent evaporation and the dried extract was collected as a powder and stored in a sealed glass vial until further use. The extraction yield was calculated according to the following Equation 4:

$$Y\;\left(\%\right)=\left(\frac{m_{2}}{m_{1}}\right)\times 100$$ (4)

Where m_1_ represents the initial mass of plant powder and m_2_ represents the mass of the dried extract obtained after evaporation.

#### 2.2.4 Dosage of phenolic compounds

##### 2.2.4.1 Determination of total polyphenol content

Total polyphenol content (TPC) of *R. tinctorum* root aqueous extract was quantified using the Folin-Ciocalteu method as described by Singleton et al. (1999). Briefly, 20 µL of the extract solution (25 mg/mL) was mixed with 1.5 mL of 10% (v/v) Folin–Ciocalteu reagent and 1.5 mL of 7.5% (w/v) sodium carbonate solution in 50 mL volumetric flasks. The mixture was diluted to volume with distilled water, thoroughly mixed, and incubated in the dark at room temperature for 40 min. Absorbance was measured at 760 nm using a UV-Vis spectrophetometer against a reagent blank (reaction mixture without extract). A gallic acid calibration curve (y = ax + b) was prepared under identical experimental conditions. TPC was calculated according to Equation 5, and the results were expressed as milligrams of gallic acid equivalents per Gram of dry extract (mg GAE/g DE).

$$TPC=\frac{C\times V_{0}}{m_{extract}}\times D$$ (5)

where C: Concentration obtained from the calibration curve (mg/mL); V_0_: Volume of the entire extract (mL); m_extract_: Mass of extract (g).

$$D=\frac{V_{f}}{V_{i}}$$

Where D: Dilution factor; V_f_: Final volume to be measured in a spectrophotometer (mL); V_i_: Volume taken from the tested extract (mL).

##### 2.2.4.2 Determination of total flavonoid content

Flavonoid content (FC) of the extract was determined using the aluminum chloride method (Chang et al., 2002). Briefly, 20 µL of the extract (25 mg/mL), 2 mL of distilled water, and 10 μL of 10% (m/V) aluminum chloride solution in methanol, were added in test tubes. The mixture was diluted to a final volume of 5 mL with pure methanol, thoroughly mixed, and incubated in the dark at room temperature for 30 min. Absorbance was measured at 433 nm against a reagent blank (reaction mixture without extract). A quercetin calibration curve (y = ax + b) was prepared under identical experimental conditions. FC was expressed as milligrams of quercetin equivalents per Gram of dry extract (mg QE/g DE), and calculated following Equation 6.

$$FC=\frac{C\times V_{0}}{m_{extract}}\times D$$ (6)

where C: Concentration obtained from the calibration curve (mg/mL); V_0_: Volume of the entire extract (mL); m_extract_: Mass of extract (g).

##### 2.2.4.3 Determination of condensed tannin content

Condensed tannin content (CTC) of the extract was evaluated using the vanillin-HCl method (Sun et al., 1998). In test tubes, 50 µL of the extract (25 mg/mL), 3 mL of 4% (m/v) vanillin solution in methanol, and 1.5 mL of 37% HCl were mixed. The mixture was thoroughly mixed and incubated in the dark at room temperature for 20 min. Absorbance was measured at 499 nm against a reagent blank (reaction mixture without extract). A catechin calibration curve (y = ax + b) was prepared under identical experimental conditions. CTC was expressed as milligrams of catechin equivalents per Gram of dry extract (mg CE/g DE) and calculated according to Equation 7.

$$CTC=\frac{C\times V_{0}}{m_{extract}}\times D$$ (7)

where C: Concentration obtained from the calibration curve (mg/mL); V_0_: Volume of the entire extract (mL); m_extract_: Mass of extract (g).

#### 2.2.5 HPLC/UV-ESI-MS analysis

Phenolic composition of the extract was analyzed using high-performance liquid chromatography coupled with electrospray ionization mass spectrometry (HPLC/UV-ESI-MS), ensuring sensitive and accurate identification of bioactive compounds. Chromatographic separation was achieved using on an UltiMate 3000 HPLC system (Thermo Fisher Scientific) equipped with a reverse-phase C18 column (Merck, Darmstadt, Germany; 250 × 4 mm, 5 μm), maintained at 40 °C. Samples were kept at 5 °C prior to injection.

The mobile phase consisted of solvent A (0.1% formic acid in water) and solvent B (0.1% formic acid in acetonitrile). Gradient elution was applied from 2% to 95% solvent B over 30 min to ensure optimal separation of phenolic constituents. The flow rate was set at 1 mL/min, and the injection volume was 20 μL.

Mass spectrometric detection was carried out in MS/MS (bbCID) mode using a Maxis Impact HD mass spectrometer (Bruker Daltonik) operating in negative ionization mode. The main operating parameters were as follows: capillary voltage, 3000 V; drying gas temperature, 200 °C; drying gas flow, 8 L/min; nebulizer pressure, 2 bar; and plate offset, −500 V. Nitrogen was used as both desolvation and nebulizing gas.

UV detection was performed using a diode array detector (Merck-Hitachi) over a wavelength range of 190–600 nm, with monitored wavelengths at 280, 320, and 360 nm corresponding to different classes of phenolic compounds. Mass spectral data were acquired over an *m/z* range of 100–1,500 and processed using Chromeleon™ 7.2 software (Thermo Scientific) for compound identification (Drioiche et al., 2023).

#### 2.2.6 Evaluation of antioxidant activity

##### 2.2.6.1 Total antioxidant capacity

Total antioxidant capacity (TAC) of the extract was determined using the phosphomolybdenum method as previously described (Hadi et al., 2024). Briefly, 5 µL of the extract solution (25 mg/mL) was mixed with 1 mL of 0.6 M sulfuric acid, 1 mL of 28 mM sodium phosphate, and 1 mL of 4 mM ammonium molybdate.

The reaction mixture was incubated at 95 °C for 90 min and then allowed to cool to room temperature for approximately 20–30 min. The absorbance was measured at 695 nm using a UV-Vis spectrophotometer. Results were expressed as milligrams of ascorbic acid equivalents per Gram of dry extract (mg AAE/g DE).

##### 2.2.6.2 DPPH test

Free radical scavenging activity of the extract was evaluated using the DPPH (2.2-diphenyl-1-picrylhydrazyl) assay according to the method described by Remok et al. (2023). Increasing volumes of the aqueous extract, previously reconstituted in distilled water, were pipetted into test tubes, and absolute ethanol was then added to obtain a volume of 200 µL. Subsequently, 2.8 mL of ethanolic DPPH solution (24 μg/mL) was added to each tube, resulting in a total reaction volume of 3 mL. The final concentration of DPPH in the reaction mixture was 22.4 μg/mL.

The mixture was incubated in the dark at room temperature for 30 min, after which the absorbance was measured at 515 nm. The percentage of DPPH radical inhibition was calculated using the following Equation 8:

$${\%}_{inhibition}=\left(\frac{A_{C}-A_{S}}{A_{C}}\right)\times 100$$ (8)

where A_C_ is the absorbance of the control and A_S_ is the absorbance of the sample.

##### 2.2.6.3 Ferric reducing antioxidant power

Ferric reducing antioxidant power (FRAP) of the extract was determined according to the method described by Saidi et al. (Moumen et al., 2025). Briefly, increasing volumes of the extract were diluted to a final volume of 0.5 mL with distilled water. The solution was mixed with 2.5 mL of 0.2 M sodium phosphate buffer (pH 6.6) and 2.5 mL of 1% potassium ferricyanide.

The mixture was incubated at 50 °C for 20 min, followed by the addition of 2.5 mL of 10% trichloroacetic acid. After centrifugation for 10 min, 2.5 mL of the supernatant was mixed with 2.5 mL of distilled water and 0.5 mL of 0.1% ferric chloride solution. The absorbance was measured at 700 nm.

#### 2.2.7 Antimicrobial activity

Antimicrobial activity of the extract was evaluated by determining the minimum inhibitory concentration (MIC) using the broth microdilution method in 96-well microplates according to a previously reported protocol (Balouiri et al., 2016).

The extract stock solution was prepared in an ethanol/distilled water mixture (30:70, v/v). Serial dilutions were performed in wells 1–10 to obtain concentrations ranging from 75 to 0.1465 mg/mL in a final volume of 100 μL of Mueller–Hinton (MH) broth for bacterial strains and Sabouraud broth for fungal strains.

Subsequently, 100 μL of microbial inoculum was added to wells 1–11 to achieve final concentrations of approximately 10^6^ CFU/mL for bacterial strains and 10^4^ CFU/mL for fungal strains. Well 11 containing broth and inoculum served as the growth control, while well 12 containing only broth served as the sterility control. Gentamicin and terbinafine were used as positive controls for antibacterial and antifungal activities, respectively, whereas the solvent (ethanol/distilled water, 30:70, v/v) was used as a negative control.

After incubation at 37 °C for 24 h, 10 μL of resazurin solution (6.75 mg/mL) was added to each well as an indicator of microbial growth, followed by further incubation for 2 h at 37 °C. A color change from blue-purple to pink indicated microbial growth. The MIC was defined as the lowest concentration of extract that prevented the color change.

To determine the minimum bactericidal concentration (MBC) and minimum fungicidal concentration (MFC), 10 μL aliquots from wells showing no visible growth was plated onto MH agar for bacterial strains or Sabouraud agar for fungal strains and incubated for 24 h at 37 °C. The MBC or MFC was defined as the lowest concentration that completely inhibited colony formation on agar plates (Prasannabalaji et al., 2012).

The antimicrobial mode of action was assessed by calculating the MBC/MIC or MFC/MIC ratio. Ratios ≤4 were considered indicative of bactericidal or fungicidal activity, whereas ratios >4 indicated bacteriostatic or fungistatic effects (National Commit tee for Clinical Laboratory Standards and Barry, 1999).

#### 2.2.8 Acute toxicity

Acute oral toxicity of the *R. tinctorum* root aqueous extract was evaluated in accordance with OECD guideline TG 423, with minor adaptations in the experimental design, including the use of mixed-sex groups (n = 6 per group) and intermediate dose levels for preliminary safety assessment (No, 2002). A total of 24 mice were used in the study. After a fasting period of 14 h, the animals were randomly divided into four groups (n = 6; ♂/♀ = 1:1).

The control group received distilled water (10 mL/kg), while the treated groups were orally administered single doses of the aqueous extract at 0.5, 1, and 2 g/kg. Body weight was recorded at the beginning of the experiment.

Following administration, the animals were continuously observed for 10 h to detect any immediate signs of toxicity, and subsequently monitored daily for 14 days for behavioral changes, clinical symptoms, or mortality. This procedure provided preliminary information regarding the safety profile of the extract at the tested doses.

#### 2.2.9 Antihyperglycemic activity

Antihyperglycemic activity of the *R. tinctorum* root aqueous extract was evaluated using the oral glucose tolerance test (OGTT) in rats, as previously described (Daoudi et al., 2020). Eighteen healthy rats were randomly divided into three groups (n = 6; ♂/♀ = 1:1): a control group receiving distilled water (10 mL/kg), a test group treated with the aqueous extract (400 mg/kg), and a reference group receiving glibenclamide (2 mg/kg). The dose of 400 mg/kg was selected as a preliminary screening dose based on previously reported pharmacological studies and supported by acute toxicity results indicating no toxicity up to 2000 mg/kg.

Baseline blood glucose levels were measured prior to treatment (t_0_). After 30 min, an oral D-glucose load (2 g/kg) was administered to all animals, and blood glucose levels were subsequently measured at 30, 60, 90, and 150 min.

This experimental protocol allowed the evaluation of the effect of the *R. tinctorum* root aqueous extract on postprandial blood glucose regulation following glucose challenge.

#### 2.2.10 Statistical analysis

Results were statistically analyzed using ANOVA (one-way analysis of variance) followed by Tukey’s *post hoc* test using GraphPad Prism software (version 9.5.1, GraphPad Prism software, San Diego, CA, United States) and they are shown as means and standard deviation. Statistics were considered to be significant at *p*-values of *p* < 0.05, *p* < 0.01, and *p* < 0.001.

#### 2.2.11 Molecular docking methodology

Molecular docking analysis was performed using AutoDock Vina to investigate the interactions between the major bioactive compounds identified in the aqueous extract of *R. tinctorum* and selected protein targets associated with the experimentally evaluated biological activities.

The three-dimensional crystal structures of the target proteins (PDB IDs: 1OG5, 3KP5, and 1B2Y) were retrieved from the Protein Data Bank. These targets were selected based on their mechanistic relevance to antioxidant, antimicrobial, and antihyperglycemic activities, respectively. Protein preparation was performed using UCSF Chimera by removing crystallographic water molecules, co-crystallized ligands, and non-essential heteroatoms. Polar hydrogen atoms were added, and Kollman charges were assigned using AutoDock Tools (version 1.5.6). The prepared protein structures were saved in PDBQT format.

The chemical structures of the selected phytochemicals were drawn using ChemDraw and converted into three-dimensional conformations. Ligand geometry optimization and energy minimization were carried out using the MMFF94 force field implemented in Open Babel before conversion to PDBQT format. Rotatable bonds were assigned to allow ligand flexibility during docking simulations.

Docking calculations were performed using AutoDock Vina with a grid box centered on the active site of each target protein. Grid dimensions were adjusted to encompass all key amino acid residues involved in ligand binding. The exhaustiveness parameter was set to ensure adequate conformational sampling. For each ligand, the best docking pose was selected based on the lowest binding affinity value (kcal/mol). Ligand–protein interactions, including hydrogen bonding, hydrophobic contacts, π–π stacking, and π–cation interactions, were analyzed and visualized using Discovery Studio Visualizer and PyMOL. Interactions with donor–acceptor distances exceeding approximately 3.5 Å were interpreted as weak hydrogen bonds or polar contacts rather than strong conventional hydrogen bonds (Filali et al., 2024; Lahyaoui et al., 2024a; Lahyaoui et al., 2024b; Sghyar et al., 2024; Sghyar et al., 2025).

## 3 Results and discussion

### 3.1 Quality control of plant material

Quality control parameters of *R. tinctorum* roots were assessed to ensure the safety, purity, and suitability of the plant for further pharmacological investigations. The results are summarized in Table 2. The moisture content was 8.92% ± 0.66%, markedly below the recommended of 12% threshold (Claudet, 2015), thereby minimizing the risk of enzymatic degradation and microbial proliferation during storage. The plant exhibited a slightly acidic pH of 6.26, confirming its acidophilic nature, and an ash content of 7.97%. Trace element analysis detected arsenic, zinc, manganese, iron, copper, and aluminum, all at concentrations below the maximum permissible limits established by FAO/WHO guidelines (Nema et al., 2014; Shirkhanloo et al., 2015).

**TABLE 2:** Moisture content, pH, ash, and heavy metals present in *Rubia tinctorum* roots.

MC %	pH	Ash (%)	Heavy metal concentration (mg/L)						
			As	Zn	Co.	Mn	Fe	Cu	Al
8.92 ± 0.66	6.26 ± 0.19	7.97 ± 0.49	0.036	0.389	ND	0.147	6.522	0.043	5.293

ND, not detected.

### 3.2 Phytochemical screening

Phytochemical screening of *R. tinctorum* roots revealed a wide range of bioactive compounds, including lipids, polysaccharides, proteins, reducing sugars, sterols and triterpenes, flavonoids, tannins, anthocyanins, leucoanthocyanins, saponosides, monosaccharides and holosides, alkaloids, reducing compounds, and mucilages. These compounds, particularly secondary metabolites, are known to exhibit a wide range of biological activities, including antioxidant, antimicrobial, anti-inflammatory, antitumor, antiseptic, hemostatic, and neuroprotective effects (Alqethami and Aldhebiani, 2021). These results are in agreement with previous reports by Houari et al., who identified saponins, tannins, quinones, phenols, free anthraquinones, flavonoids, alkaloids, cardiac glycosides, and proteins in the hydromethanolic extract of *R. tinctorum* roots (Zohra et al., 2022). Additionally, the hydroethanolic Soxhlet extract was found to be rich in flavonoids, tannins, anthocyanins, quinones, and catechols (Marhoume, 2021).

### 3.3 Extraction yield and contents of total polyphenols, flavonoids and condensed tannins

Results of extraction yield and total polyphenol, flavonoid, and condensed tannin contents of *R. tinctorum* root aqueous extract are summarized in Table 3. The extract provided a yield of 19.05% ± 0.74%. Notably, the yield of the hydroethanolic Soxhlet extract was previously reported as 12.75% by Marhoume et al. (Zohra et al., 2022).

**TABLE 3:** Extraction yields and total polyphenol, flavonoid, and condensed tannin contents of *Rubia tinctorum* root aqueous extract.

Extract	Extraction yield (%)	Total polyphenols (mg GAE/g DE)	Flavonoids (mg QE/g DE)	Condensed tannins (mg CE/g DE)
Aqueous	19.05 ± 0.74	40.58 ± 1.01	9.43 ± 0.81	11.96 ± 0.45

Mean values ± standard deviations of determinations performed in triplicate are reported. Means are significantly different (p < 0.05).

Regarding phenolic composition, the extract contains 40.58 ± 1.01 mg GAE/g DE, 9.43 ± 0.81 mg QE/g DE, 11.96 ± 0.45 mg CE/g DE of total polyphenols, flavonoids, and condensed tannins, respectively. Compared with previously published data, Marhoume et al. reported a total polyphenol content of 18.37 ± 0.58 mg GAE/g DE in the hydroethanolic Soxhlet extract (Marhoume et al., 2021). Flavonoid and condensed tannin contents were also lower in previous reports (2.4 ± 0.16 mg CE/g DE and 2.0 ± 0.10 mg CE/g DE, respectively), despite the use of similar extraction procedures. These differences may be attributed to variations in extraction method, solvent polarity, plant origin, maturity, harvest time, pre-treatment, and experimental conditions, such as temperature and extraction duration, which can strongly affect phenolic concentrations.

These phenolic constituents may partly account for the pharmacological activities attributed to *R. tinctorum* roots. Polyphenols, as natural antioxidants, have been associated with protection against cancer, cardiovascular disorders, diabetes, and neurological diseases (Aguilera et al., 2016; Pandey and Rizvi, 2009). Flavonoids, the second largest group of dietary polyphenols, are abundant in a variety of plant-based foods and have been investigated for potential hypoglycemic effects (Li et al., 2023; Rees et al., 2018). Condensed tannins, also known as proanthocyanidins, are formed through flavanol condensation and are present in flowers, fruits, leaves, and seeds (De la Iglesia et al., 2010; Rauf et al., 2019). These compounds contribute to plant defense and exhibit antioxidant, antimicrobial, anticancer, antidiabetic, and neuroprotective activities (Rauf et al., 2019).

### 3.4 Chemical composition of the aqueous extract of *Rubia tinctorum* roots

**FIGURE 2:**
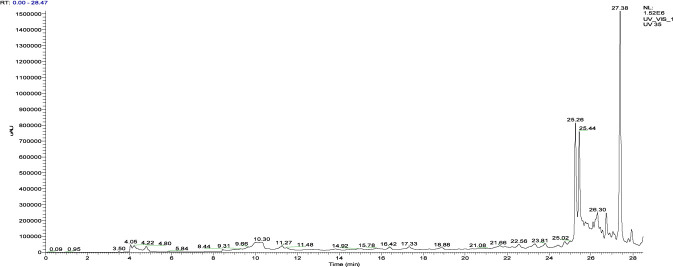
HPLC chromatogram of the aqueous extract of *Rubia tinctorum* roots.

HPLC/UV-ESI-MS analysis of the extract led to the identification of 42 compounds, as summarized in Table 4. Chromatographic profile shown in Figure 2 highlights the peaks detected in the extract. Compounds were identified based on their chromatographic behavior and mass spectrometric data. Analysis revealed the presence of several classes of secondary metabolites, including phenolic acids, flavonoids, anthraquinones, anthraquinone glycosides, quinones, lignans, and coumarins, which together accounted for 99.2% of the total composition of the extract.

**TABLE 4:** Chemical composition of the aqueous extract of *Rubia tinctorum* roots.

N°	Rt (min)	Area (%)	(m/z) [M-H]^–^	(m/z) [M-H]^+^	MW	Identified compound	Molecular formula	Class
1	4.05	0.53	-	155	154	Protocatechuic acid	C_7_H_6_O_4_	Phenolic acid
2	4.22	0.86	-	365	364	Harpagide	C_15_H_24_O_10_	Iridoid
3	4.38	0.52	-	317	316	Rhamnetin	C_16_H_12_O_7_	Flavonoid
4	4.80	0.82	367	-	368	Feruloylquinic acid	C_17_H_20_O_9_	Phenolic acid
5	8.99	0.47	-	147	146	Coumarin	C_9_H_6_O_2_	Coumarin
6	9.31	0.48	-	409	408	3-O-Methylniveusin A	C_21_H_28_O_8_	Flavonoid
7	9.66	0.77	-	197	196	Hydroxycaffeic acid	C_9_H_8_O_5_	Phenolic acid
8	10.01	2.13	-	241	240	Alizarin	C_14_H_8_O_4_	Anthraquinone
9	10.30	2.00	-	257	256	Purpurin	C_14_H_8_O_5_	Anthraquinone
10	10.62	0.38	-	301	300	Pseudopurpurin	C_15_H_8_O_7_	Anthraquinone
11	11.27	1.16	-	345	344	Cirsileneol	C_18_H_16_O_7_	Flavonoid
12	11.48	0.54	-	451	450	Phylloquinone	C_31_H_46_O_2_	Quinone
13	16.35	1.5	-	199	198	Syringic acid	C_9_H_10_O_5_	Phenolic acid
14	17.33	0.68	-	477	476	Cirsimarin	C_23_H_24_O_11_	Flavonoid
15	17.41	0.62	401	-	402	Rubianine	C_20_H_18_O_9_	Anthraquinone
16	18.88	0.56	-	433	432	Lucidin 3-O-glucoside	C_21_H_20_O_10_	Anthraquinone glycoside
17	19.87	0.72	-	521	520	Methyl 4-O-galloylchlorogenate	C_24_H_24_O_13_	Phenolic acid
18	21.08	0.58	-	565	564	Aloinoside B	C_27_H_32_O_13_	Anthraquinone glycoside
19	21.3	1.2	-	363	362	Secoisolariciresinol	C_20_H_26_O_6_	Lignan
20	21.66	1.46	-	609	608	Diosmin	C_28_H_32_O_15_	Flavonoid
21	22.25	0.60	-	357	356	Caffeic acid 4-O-glucuronide	C_15_H_16_O_10_	Phenolic acid
22	22.33	0.66	207	-	208	Anthraquinone	C_14_H_8_O_2_	Anthraquinone
23	22.56	1.48	539	-	540	Yunnaneic acid D	C_27_H_24_O_12_	Phenolic acid
24	23.32	2.05	-	171	170	Gallic acid	C_7_H_6_O_5_	Phenolic acid
25	23.81	1.87	-	255	254	Alizarin 1-methyl ether	C_15_H_10_O_4_	Anthraquinone
26	24.43	1.70	-	345	344	Rivularin	C_18_H_16_O_7_	Anthraquinone
27	24.75	1.48	-	433	432	Emodin 8-glucoside	C_21_H_20_O_10_	Anthraquinone glycoside
28	25.02	1.38	387	-	388	Ethyl rosmarinate	C_20_H_20_O_8_	Phenolic acid
29	25.26	10.74	593	-	594	Galiosin	C_26_H_26_O_16_	Anthraquinone glycoside
30	25.44	10.09	253	-	254	Rubiadin	C_15_H_10_O_4_	Anthraquinone
31	25.61	3.08	-	251	252	Methoxy-2-methylanthraquinone	C_16_H_12_O_3_	Anthraquinone
32	25.73	1.91	-	331	330	Kermesic acid	C_16_H_10_O_8_	Anthraquinone
33	25.84	3.01	-	565	564	Lucidin-3-O-primeveroside	C_26_H_28_O_14_	Anthraquinone glycoside
34	26.00	1.05	-	179	178	Methyl p-coumarate	C_10_H_10_O_3_	Phenolic acid
35	26.09	2.20	-	459	458	Epigallocatechin gallate	C_22_H_18_O_11_	Flavonoid
36	26.30	6.02	491	-	492	Salvianolic acid C	C_26_H_20_O_10_	Phenolic acid
37	26.44	1.88	-	417	416	Apigenin 7-rhamnoside	C_21_H_20_O_9_	Flavonoid
38	26.55	1.78	-	355	354	Chlorogenic acid	C_16_H_18_O_9_	Phenolic acid
39	26.73	3.72	-	463	462	Pseudopurpurin glucoside	C_21_H_18_O_12_	Anthraquinone glycoside
40	26.89	1.75	-	289	288	Dihydrokaempferol	C_15_H_12_O_6_	Flavonoid
41	27.05	2.93	385	-	386	Arctigenin methyl ether	C_22_H_26_O_6_	Lignan
42	27.38	19.84	533	-	534	Ruberythric acid	C_25_H_26_O_13_	Anthraquinone glycoside

**FIGURE 3:**
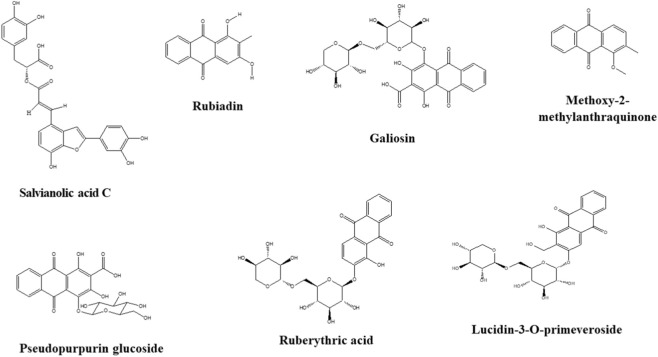
Structures of the major compounds identified in the aqueous extract of *Rubia tinctorum* roots.

Seven compounds exhibited a relative abundance ≥3%, namely, ruberythric acid (19.84%), galiosin (10.74%), rubiadin (10.09%), salvianolic acid C (6.02%), pseudopurpurin glucoside (3.72%), methoxy-2-methylanthraquinone (3.08%), and lucidin-3-O-primeveroside (3.01%). Figure 3 illustrates the chemical structures of the major compounds identified in the aqueous root extract of *R. tinctorum*.

Anthraquinone derivatives, which represent characteristic constituents of *Rubia tinctorum* species, are known to exhibit a wide range of pharmacological activities, including antioxidant, anti-inflammatory, antibacterial, anticancer, and antiplatelet effects (Wang et al., 2021). Among the identified compounds, ruberythric acid, a glycosylated anthraquinone derivative present in the roots of *R. tinctorum* L., is considered one of the major biologically active constituents of this species (Vasil’ev et al., 2024). Galiosin, identified in *Rubia* species, was first isolated by Hill and Richter in 1936 and has been suggested to act as a precursor of purpurin in madder dye (Cooksey, 2020). Rubiadin, a bioactive anthraquinone exhibits antioxidant, nephroprotective, hepatoprotective, and immunomodulatory activities (Chitsaz et al., 2021; Derksen, 2001).

Salvianolic acid C is a hydrophilic phenolic compound known for its antioxidant, anti-inflammatory, and anti-apoptotic activities, as well as its ability to inhibit SARS-CoV-2 infection (Su et al., 2025; Tang and Zhao, 2019; Yang et al., 2020). Alizarin, one of the most widely studied anthraquinones from *R. tinctorum*, exhibits antibacterial, antitumor, antioxidant, analgesic, and anti-inflammatory activities (Derksen, 2001; Humbare et al., 2022; Wang et al., 2021), while purpurin demonstrates similar pharmacological effects (Humbare et al., 2022; Wang et al., 2021). Alizarin-1-methyl ether has been reported to possess anti-HIV, cytotoxic, and antimicrobial activities (Ali et al., 2000).

In addition, chlorogenic acid is known for its antibacterial, anti-inflammatory, antimutagenic, antioxidant, cardioprotective, and anticancer properties (Lou et al., 2011; Olthof et al., 2001). Dihydrokaempferol acts as a potent antioxidant and has been reported to play a role in the treatment of severe acute pancreatitis (Liang et al., 2020). Diosmin is widely used in clinical practice for the management of chronic venous disorders and hemorrhoids due to its anti-inflammatory effects (Cazaubon et al., 2021; Rahman et al., 2024). Ethyl rosmarinate has been associated with vascular protective, antioxidant, anti-inflammatory, and antidiabetic activities (Shen et al., 2018). Protocatechuic acid exhibits a broad spectrum of pharmacological effects, including antioxidant, anti-inflammatory, neuroprotective, antibacterial, antiviral, anticancer, antidiabetic, antiproliferative, and cardioprotective properties (Cadena-Iñiguez et al., 2024; Song et al., 2020; Tanaka et al., 2011).

The predominance of anthraquinone derivatives in the extract agrees with previous reports describing *R. tinctorum* roots as an important source of anthraquinone-based pigments, including lucidin primeveroside, ruberythric acid, galiosin, rubiadin primeveroside, alizarin, purpurin, pseudopurpurin, lucidin, and xanthopurpurin (Cuoco et al., 2009). Other anthraquinones such as munjistin, nordamnacanthal, anthragallol have also been previously reported in this species (Derksen, 2001; Mouri and Laursen, 2012).

### 3.5 Antioxidant activities

Antioxidant activity of *R. tinctorum* root aqueous extract was evaluated using TAC, DPPH radical scavenging, and ferric reducing antioxidant power (FRAP) assays (Table 5). Extract exhibited a TAC of 165.26 ± 0.46 mg AAE/g DE, a DPPH IC_50_ of 265.17 ± 0.68 μg/mL, and a FRAP EC_50_ of 147.04 ± 0.71 μg/mL, indicating measurable electron-donating and radical scavenging capacity. Decoction method likely enhanced extraction of highly polar antioxidant compounds. Ascorbic acid, used as reference, showed stronger activity (DPPH IC_50_ = 6.39 ± 0.61 μg/mL; FRAP EC_50_ = 6.27 ± 0.72 μg/mL), validating the robustness of the antioxidant assay.

**TABLE 5:** Antioxidant activities of *Rubia tinctorum* root aqueous extract.

Extract	TAC (mg AAE/g DE)	DPPH* IC_50_ (µg/mL)	FRAP EC_50_ (µg/mL)
Aqueous	165.26 ± 0.46	265.17 ± 0.68	147.04 ± 0.71
Ascorbic acid	-	6.39 ± 0.61	6.27 ± 0.72

Mean values ± standard deviations of determinations performed in triplicate are reported. Means are significantly different (p < 0.05).

HPLC/UV-MS-ESI analysis identified phenolic and anthraquinone compounds, including ruberythric acid, galiosin, rubiadin, salvianolic acid C, chlorogenic acid, and syringic acid, which likely contributing to the observed antioxidant activity.

TAC reflects overall electron-donating capacity, DPPH assay evaluates free radical neutralization, and FRAP method measures reduction of Fe^3+^ to Fe^2+^ (Biskup et al., 2013; Jan et al., 2013; Kedare and Singh, 2011; Pop et al., 2015).

Houari et al. reported a TAC of 17.23 ± 0.009 mg/mL for a hydromethanolic extract of *R. tinctorum* roots (Zohra et al., 2022). Marhoume et al. described an IC_50_ value of 156.44 ± 35.76 μg/mL and an EC_50_ value of 2.44 ± 0.02 μg/mL for a hydroethanolic Soxhlet extract (Marhoume et al., 2021).

### 3.6 Antimicrobial activity

Antimicrobial potential of the aqueous extract was evaluated against bacterial and fungal strains (Table 6). MIC values ranged from 9.38 mg/mL (*Escherichia coli*, *P. mirabilis*, *C. albicans*, *C. krusei*, *C. parapsilosis*, *C. tropicalis*, *C. dubliniensis*, *S. cerevisiae*, *Aspergillus niger*) to 75 mg/mL (*S. epidermidis*, *Streptococcus agalactiae*, *E. coli* ESBL, *P. aeruginosa*, *Enterobacter cloacae*), indicating moderate broad-spectrum inhibitory activity. Standard controls, gentamicin and terbinafine, exhibited lower MICs, confirming their higher antimicrobial potency compared to the plant extract.

**TABLE 6:** MIC, MBC, and MFC (mg/mL) values of *Rubia tinctorum* root aqueous extract, along with the MIC values of reference antibiotic and antifungal agents.

Microorganism	Aqueous extract			Gentamicin	Terbinafine
	MIC	MBC or MFC	Ratio	MIC	MIC
*S. epidermidis*	75	>75	-	2	​
*S. aureus* BLACT	37.5	>75	-	<0.5	​
*S. agalactiae* (B)	75	>75	-	≤250	​
*E. coli* ESBL	75	>75	-	2	​
*K. pneumonie*	18.75	>75	-	≤1	​
*E. coli*	9.38	>75	-	2	​
*P. mirabilis*	9.38	>75	-	2	​
*P. aeruginosa*	75	>75	-	2	​
*E. cloacae*	75	>75	-	>4	​
*C. albicans*	9.38	>75	-	​	12.50
*C. kefyr*	18.75	37.5	2	​	25
*C.krusei*	9.38	75	8	​	50
*C. parapsilosis*	9.38	>75	-	​	6.25
*C. tropicalis*	9.38	75	8	​	12.50
*C. dubliniensis*	9.38	>75	-	​	3.13
*S. cerevisiae*	9.38	37.5	4	​	3.13
*A. niger*	9.38	9.38	1	​	3.13

Analysis of MBC/MIC and MFC/MIC ratios revealed that no bactericidal activity was observed against the tested bacterial strains within the concentration range evaluated, as all MBC values exceeded the highest concentration tested (75 mg/mL). For fungi, fungicidal effects were detected against *C. kefyr*, *S. cerevisiae*, and *A. niger* (MFC/MIC ≤4), while fungistatic effects were observed against *C. krusei* and *C. tropicalis* (MFC/MIC = 8). For the remaining fungal strains, MFC values exceeded the highest concentration tested, precluding a definitive classification based on the MFC/MIC ratio.

Overall, the aqueous extract showed inhibitory activity against both bacterial and fungal strains, with a more pronounced effect on fungi, suggesting a differential antimicrobial profile and warranting further phytochemical investigation.

Previous studies reported antimicrobial activity of a methanolic extract of *R. tinctorum* roots against *Klebsiella pneumoniae* and *S. aureus* (MIC = 3.125 mg/mL), but weaker effects against *E. coli* and *P. aeruginosa* (MIC = 25 mg/mL) (El Tanahy, 2022). These differences are likely related to the extraction solvent and resulting phytochemical composition. Similarly, inhibitory activity against *C. albicans* and *S. cerevisiae* has been demonstrated by aqueous infusion of *R. tinctorum* roots (Kalyoncu et al., 2006).

### 3.7 Acute toxicity

Oral administration of medicinal and aromatic plants can sometimes induce toxic effects; therefore, an *in vivo* acute toxicity study was performed using aqueous extract of *R. tinctorum* roots. A single dose of 2 g/kg did not produce any observable signs of toxicity, including diarrhea, vomiting, abnormal mobility, or mortality, throughout the monitoring period, indicating that aqueous extract is well tolerated at this dose.

These findings are consistent with previous reports, in which oral administration of *R. tinctorum* roots to male and female mice at doses ranging from 0 to 5,000 mg/kg for 14 days was well tolerated. No adverse effects were observed at doses up to 3,500 mg/kg, whereas a single mortality occurred in the 5,000 mg/kg group, likely due to difficulties in administration. No clinical abnormalities or specific pathological lesions were observed, confirming that *R. tinctorum* roots exhibit no significant acute toxicity in mice (Ino et al., 1995).

### 3.8 Antihyperglycemic effect

**FIGURE 4:**
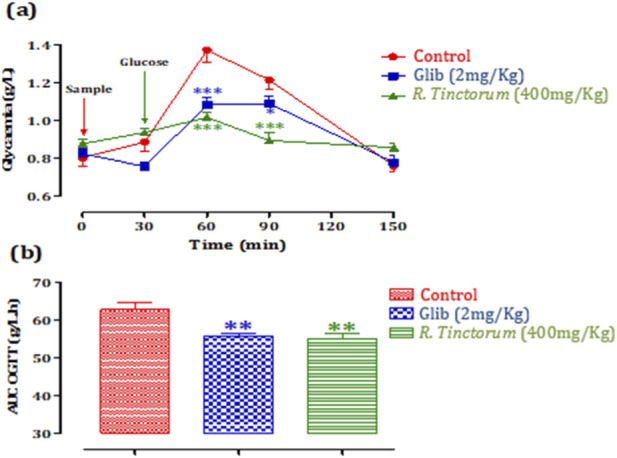
Postprandial blood glucose **(a)** and area under the postprandial curve **(b)** in normal rats after administration of *Rubia tinctorum* root aqueous extract and glibenclamide. Values are mean ± SEM (n = 6). ***p < 0.001; **p < 0.01; *p < 0.05: compared to the control.

As shown in Figure 4, normal rats exhibited a marked increase in blood glucose levels 30 min after oral glucose administration. Pretreatment with aqueous extract of *R. tinctorum* roots (400 mg/kg) or glibenclamide (2 mg/kg) significantly improved glycemic response. Oral administration of aqueous extract 30 min prior to glucose loading significantly reduced postprandial hyperglycemia at 60 and 90 min, similar to glibenclamide. Both treatments produced comparable glycemic profiles, and area under the glucose curve (AUC) was significantly decreased relative to distilled water-pretreated control group, indicating antihyperglycemic potential of aqueous extract.

A limitation of the present study is that only a single dose of the extract was evaluated, which does not allow assessment of dose-response relationships. Further studies using multiple doses are required to better characterize the antihyperglycemic potential of the extract.

### 3.9 Molecular docking

The major phytochemicals identified in the aqueous extract, including lucidin-3-O-primeveroside, galiosin, rubiadin, salvianolic acid C, methoxy-2-methylanthraquinone, pseudopurpurin, and ruberythric acid, were subjected to molecular docking studies against three protein targets associated with the experimentally investigated biological activities, namely, antioxidant, antimicrobial, and antihyperglycemic effects. The selected protein structures, retrieved from the Protein Data Bank, included 1OG5 (human glutathione S-transferase Pi, GST Pi), 3KP5 (a LuxR-type bacterial quorum-sensing regulatory protein from *Vibrio* harveyi), and 1B2Y (human pancreatic α-amylase). The 1OG5 target was selected as an oxidative stress- and detoxification-related protein involved in cellular antioxidant defense mechanisms, in order to provide complementary mechanistic insight into the antioxidant potential of the identified phytochemicals. Similarly, 3KP5 was used as a representative bacterial regulatory target associated with quorum sensing, microbial communication, and pathogenicity. However, this target does not specifically represent all experimentally tested microorganisms and was therefore used as a complementary mechanistic model rather than a universal antimicrobial target. Although these docking targets were selected based on their mechanistic relevance, the obtained results remain indicative and require further experimental and biological validation.

#### 3.9.1 Antioxidant activity

**FIGURE 5:**
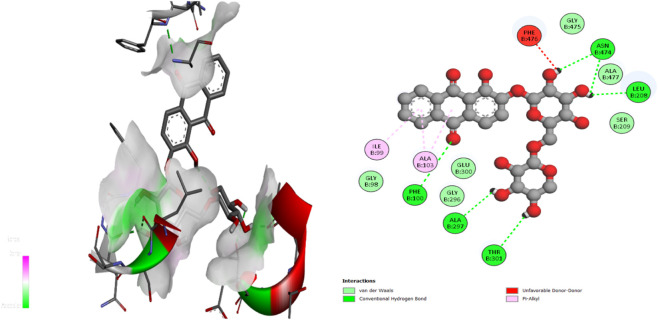
Docking of ruberythric acid and 1OG5 protein.

Molecular docking analysis provided additional insight into the potential contribution of the major phenolic and anthraquinone compounds identified in the aqueous extract to the antioxidant activity observed *in vitro*. The binding affinities of the investigated compounds toward the antioxidant target protein (1OG5) ranged from −5.92 to −11.10 kcal/mol. Among all tested molecules, ruberythric acid exhibited the strongest binding affinity (−11.10 kcal/mol), followed by salvianolic acid C (−9.90 kcal/mol) and lucidin-3-O-primeveroside (−9.13 kcal/mol) (Table 7; Figure 5). The remarkably low binding energy obtained for ruberythric acid suggests the formation of a highly stable ligand–protein complex and highlights its potential contribution to the antioxidant properties of the extract.

**TABLE 7:** Docking score results of the studied proteins.

Compound	Binding energy (Kcal/mol)		
	1OG5	3KP5	1B2Y
Methoxy-2-methylanthraquinone	−6.57	−4.79	−4.82
Galiosin	−7.45	−6.45	−7.76
Lucidin-3-O-primeveroside	−9.13	−6.3	−7.48
Pseudopurpurin	−6.66	−5.53	−6.44
Rubiadin	−5.92	−4.8	−5.03
Ruberythric acid	−11.1	−10.6	−10.3
Salvianolic acid C	−9.9	−6.65	−7.33

Detailed interaction analysis revealed that ruberythric acid established several conventional hydrogen bonds with key amino acid residues, including ASN474, LEU208, PHE100, ALA297, and THR301, which contribute significantly to complex stabilization. Additional π–alkyl interactions were observed with ILE99 and ALA103, strengthening hydrophobic contacts within the binding pocket. Furthermore, surrounding residues such as GLY98, GLY296, GLU300, GLY475, ALA477, and SER209 participated through van der Waals interactions, providing additional stabilization of the docked conformation. An unfavorable donor–donor interaction with PHE476 was also detected; however, its effect appeared insufficient to offset the numerous favorable interactions responsible for the strong binding affinity.

In contrast, rubiadin displayed the weakest interaction profile (−5.92 kcal/mol), indicating a comparatively lower contribution to the antioxidant potential of the extract. The superior docking performance of ruberythric acid, together with the favorable binding energies of salvianolic acid C and lucidin-3-O-primeveroside, suggests that these compounds may represent key contributors to the antioxidant activity experimentally observed for the aqueous extract of *R. tinctorum*.

Overall, the docking results provide complementary mechanistic support for the antioxidant assays and indicate that ruberythric acid is the most promising antioxidant-related ligand among the identified phytochemicals, owing to its extensive hydrogen-bonding network and multiple hydrophobic interactions within the active site of the target protein.

#### 3.9.2 Antimicrobial activity

**FIGURE 6:**
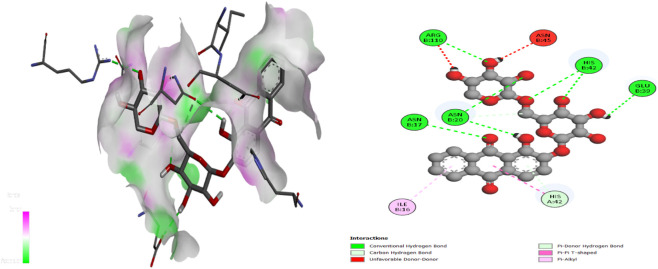
Docking of ruberythric acid and 3KP5 protein.

Molecular docking analysis against the antimicrobial target protein 3KP5 provided complementary insight into the potential mechanisms underlying the antimicrobial activity of *R. tinctorum* root aqueous extract. Among the investigated compounds, ruberythric acid exhibited the strongest binding affinity, with a docking score of −10.60 kcal/mol (Table 7; Figure 6), substantially exceeding those of the other identified phytochemicals. This remarkably low binding energy indicates the formation of a highly stable protein–ligand complex and suggests a significant contribution of ruberythric acid to the antimicrobial potential of the extract.

Detailed interaction analysis revealed that ruberythric acid formed an extensive network of conventional hydrogen bonds with Asn17, Asn20, His42, Glu39, and Arg110, which play a major role in stabilizing the ligand within the binding cavity. Additional carbon hydrogen-bond interactions involving Asn20 and His42 further reinforced ligand accommodation and contributed to the overall binding stability. Moreover, the anthraquinone aromatic scaffold established a π–π T-shaped interaction with His42 and a π–alkyl interaction with Ile16, providing additional hydrophobic stabilization of the complex.

Although unfavorable donor–donor interactions were detected with Asn45 and Arg110, these contacts did not appear to significantly compromise the overall binding pattern, as evidenced by the exceptionally favorable binding energy. The predominance of stabilizing hydrogen-bond and hydrophobic interactions suggests that ruberythric acid can effectively occupy the active site of the target protein and potentially interfere with biological processes associated with microbial virulence and survival.

Compared with the other investigated compounds, ruberythric acid demonstrated a markedly superior binding profile, highlighting its potential role as one of the principal antimicrobial constituents of the extract. These findings are consistent with the experimental antimicrobial activity observed against several bacterial and fungal strains and provide a molecular basis for the bioactivity of *R. tinctorum* root aqueous extract. Collectively, the docking results suggest that ruberythric acid may contribute significantly to the antimicrobial properties of the extract through strong and stable interactions with microbial regulatory proteins.

The docking analysis provides complementary mechanistic insight into the possible antimicrobial-related interactions of the identified phytochemicals, particularly through quorum-sensing-associated pathways. Nevertheless, further docking investigations using pathogen-specific molecular targets are required to establish a stronger correlation between the *in silico* findings and the experimentally tested microbial strains.

#### 3.9.3 Antihyperglycemic activity

**FIGURE 7:**
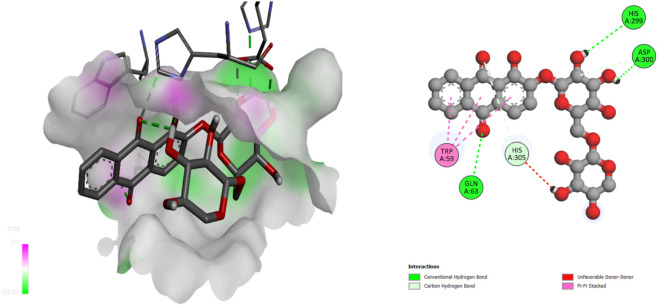
Docking of ruberythric acid and 1B2Y protein.

Molecular docking analysis against the antihyperglycemic target protein 1B2Y revealed that ruberythric acid exhibited the strongest binding affinity among all investigated compounds, with a docking score of −10.30 kcal/mol (Table 7; Figure 7). This highly favorable binding energy indicates the formation of a stable protein–ligand complex and suggests a strong potential for interaction with residues located within the enzyme active site. The superior binding profile of ruberythric acid compared with the other identified phytochemicals highlights its potential contribution to the antihyperglycemic activity of the aqueous extract.

Detailed examination of the docking pose demonstrated that ruberythric acid established several conventional hydrogen bonds with Gln63, His299, and Asp300, while an additional carbon hydrogen-bond interaction was observed with His305. These interactions contribute significantly to ligand stabilization within the catalytic cavity and promote favorable positioning of the molecule for efficient receptor recognition. The presence of multiple hydroxyl groups and glycosidic moieties in the ruberythric acid structure enables the formation of an extensive hydrogen-bonding network, thereby enhancing both binding affinity and specificity toward the target protein.

In addition to hydrogen-bond interactions, the aromatic anthraquinone scaffold of ruberythric acid formed multiple π–π stacked interactions with Trp59, a residue known to play an important role in substrate recognition and stabilization within carbohydrate-metabolizing enzymes. These aromatic interactions provide further stabilization of the ligand–protein complex through favorable electronic and hydrophobic contacts. Although an unfavorable donor–donor interaction with His305 was detected, its effect appears minimal when compared with the numerous stabilizing interactions responsible for the excellent docking score.

The strong interaction pattern observed with Asp300, Gln63, His299, His305, and Trp59 suggests that ruberythric acid may interfere with the catalytic activity of the target enzyme and thereby contribute to the regulation of glucose metabolism. These findings are consistent with the *in vivo* oral glucose tolerance test, in which the aqueous extract significantly reduced postprandial blood glucose levels. Collectively, the docking and biological results support the hypothesis that ruberythric acid is one of the major contributors to the antihyperglycemic activity of *R. tinctorum* roots and represents a promising natural lead compound for the development of novel antidiabetic agents.

## 4 Conclusion

The present study provides a comprehensive phytochemical and biological evaluation of *Rubia tinctorum* L. root aqueous extract. Chemical composition analysis, acute toxicity assessment, antihyperglycemic activity, and computational studies were performed, highlighting its remarkable pharmacological potential. The extract contained total polyphenols, flavonoids, and condensed tannins, compounds commonly associated with antioxidant and antimicrobial properties. HPLC/UV-ESI-MS analyses confirmed that the extract was particularly rich in phenolic and anthraquinone derivatives, including ruberythric acid, galiosin, rubiadin, and salvianolic acid C—compounds known for their strong antioxidant and antimicrobial properties.

*In vivo* evaluations demonstrated significant antihyperglycemic activity and confirmed safety in the acute toxicity study. Integration of *in vitro*, *in vivo*, and *in silico* approaches provided mechanistic insights into these bioactivities. Molecular docking analyses suggested favorable interactions between major anthraquinones and proteins associated with oxidative stress regulation, microbial resistance, and carbohydrate metabolism, supporting the molecular basis for the experimentally observed effects.

Overall, the aqueous extract of *R. tinctorum* roots may be considered a preliminary source of bioactive compounds, contributing to the valorization of Moroccan medicinal flora and supporting further investigation in evidence-based natural product research. Future investigations should focus on bioassay-guided isolation of active constituents, detailed pharmacological profiling, and exploration of potential synergistic interactions among the identified compounds to further elucidate their underlying mechanisms of action.

## Data Availability

The original contributions presented in the study are included in the article/supplementary material, further inquiries can be directed to the corresponding author.
